# Clinical Use of Optical Coherence Tomography Angiography in Retinal Diseases

**DOI:** 10.3390/diagnostics13101820

**Published:** 2023-05-22

**Authors:** Figen Batıoğlu, Özge Yanık, Sibel Demirel, Emin Özmert

**Affiliations:** Department of Ophthalmology, Ankara University School of Medicine, 06620 Ankara, Turkey; oyanik05@hotmail.com (Ö.Y.); drsibeldemireltr@yahoo.com.tr (S.D.); eozmert56@gmail.com (E.Ö.)

**Keywords:** age-related macular degeneration, macular neovascularization, optical coherence tomography angiography, pachychoroid neovasculopathy, polypoidal choroidal vasculopathy, diabetic retinopathy, retinal vein occlusions, retinal artery occlusion, idiopathic macular telangiectasia type 2

## Abstract

The advent of optical coherence tomography angiography (OCTA) is one of the cornerstones of fundus imaging. Essentially, its mechanism depends on the visualization of blood vessels by using the flow of erythrocytes as an intrinsic contrast agent. Although it has only recently come into clinical use, OCTA has become a non-invasive diagnostic tool for the diagnosis and follow-up of many retinal diseases, and the integration of OCTA in multimodal imaging has provided a better understanding of many retinal disorders. Here, we provide a detailed overview of the current applications of OCTA technology in the diagnosis and follow-up of various retinal disorders.

## 1. Introduction

Optical coherence tomography angiography (OCTA) is a new, non-invasive imaging modality that provides high-quality images of the retinal and choroidal vascular structures [1]. Although it is still a developing technology, the reliability and reproducibility of its measurements have been proven [2]. The most important advantage of the system is its capability to display functional and structural data in simultaneous sections [1,2].

The basic working principle of OCTA is based on the detection of contrast created by the movement of erythrocytes on a stationary background. The algorithms evaluate the signal differences between repeating sections passing through the same location. The signal difference between two cross-sectional B-scan OCT images, taken consecutively from a single point on the retina, is calculated. Then, colored flow signals are superimposed on the B-scan OCT images, and three-dimensional OCTA images (OCTA cube) are created.

Commercially available OCTA devices use swept-source (SS-OCTA) or spectral domain (SD-OCTA) systems. SS-OCTA devices have a wavelength of 1050 nm, providing axial scan rates of up to 100–236 kHz A-scans per second, while traditional SD-OCTA systems use a wavelength of 840 nm, acquiring an approximately 70–85 kHz A-scan rate [3,4]. The longer wavelength of SS-OCTA devices enables deeper tissue penetration of the light, providing improved imaging of the choroid and choriocapillaris with less scattering and interference.

**Figure 1:**
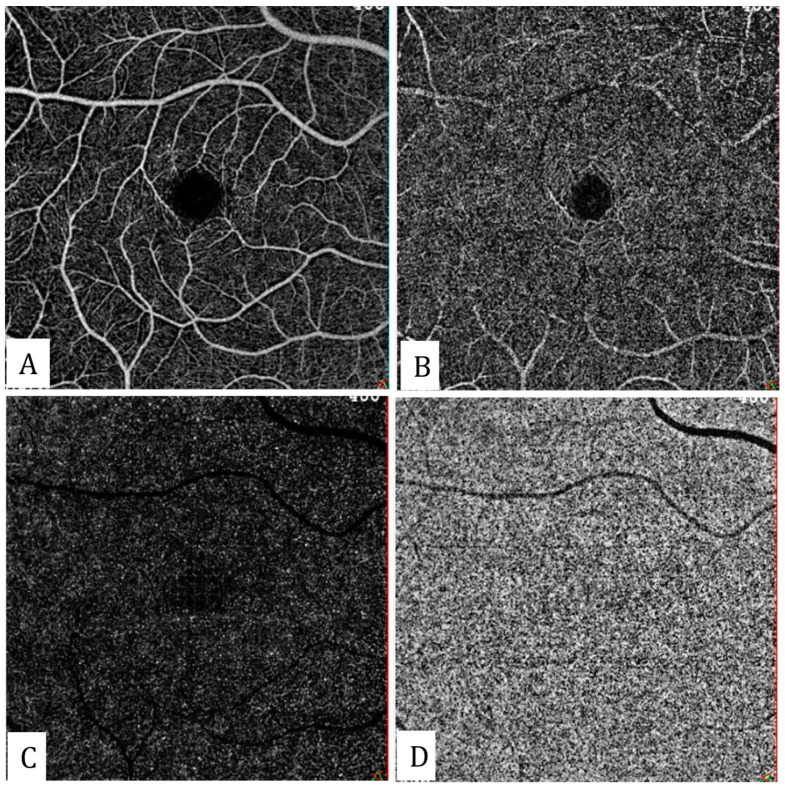
Autosegmented en-face OCTA slabs: (**A**) superficial capillary plexus, (**B**) deep capillary plexus, (**C**) outer retina, and (**D**) choriocapillaris.

The auto-segmentation feature of the software can generate OCTA slabs passing through different layers of the retina and choroid, and the segmentation level can also be adjusted manually to avoid segmentation errors. These slabs basically include the superficial capillary plexus, deep capillary plexus, the outer retina, and choriocapillaris (Figure 1).

Nowadays, OCTA has become a non-invasive diagnostic tool for the diagnosis and follow-up of many retinal diseases. In this section, OCTA findings in various retinal diseases will be discussed.

### Limitations of OCTA and Imaging Artefacts

The most significant limitation of OCTA is that, unlike conventional dye angiographies, it is not capable of detecting vascular leakage, which is an important sign of blood–retinal barrier breakdown. In fact, this feature provides more prominent visualization of vascular networks, with their exact boundaries. However, sometimes it turns into a disadvantage, especially with regard to inflammatory vascular pathologies, determination of the lesion activity, or differentiation of neovascularization and microvascular anomalies. In addition, although some devices are able to achieve a wider scan, it is still not possible to provide ultra-wide field retinal imaging with currently available OCTA systems.

Among the OCTA artifacts, the most well-known are low-intensity artifacts, motion artifacts, projection artifacts, shadowing artifacts, false positive and false negative flow artifacts, and segmentation errors.

Due to the reflection of the vibrational signals passing through the large blood vessels of the retina from the RPE/photoreceptor layer, a copy image of these large retinal vessels may be formed in the deep layers of the retina and is known as the “projection artifact”. With the development of software updates for projection artefact removal and motion-correction and dual tracking, these artifacts could be partially ruled out. However, in this case, post-processing artifacts may develop due to the intervention of the device to reduce the artifacts.

Since OCTA has lower and upper threshold values for flow detection, it may not be able to detect reduced flows below its threshold limit or high turbulent flows above its threshold value. The inability to detect reduced flow is known as the false negative flow artifact, while the inability to show rapid flow in the large choroid vessels is known as the “fringe wash-out” artifact. Additionally, the suspended scattering particles in motion is a newly described artifact which develops due to non-erythrocyte moving particles.

## 2. Clinical Applications

### 2.1. Age-Related Macular Degeneration

Histopathological studies of age-related macular degeneration have revealed significant loss of choriocapillaris, even in the early stages of the disease [5]. OCTA technology has further strengthened this existing knowledge.

In non-neovascular age-related macular degeneration (AMD), it has been shown that the vascular density of the choriocapillaris decreases with increased drusen density [6]. In fact, this change is not limited to the choriocapillaris slab alone, but it has been shown to affect all choroidal layers [7]. Especially in the presence of reticular pseudodrusen, the loss of choriocapillaris signal in OCTA is higher than those in other drusen types [8]. In a study comparing SS-OCTA and SD-OCTA features under drusen, a 30.4% signal loss was observed in SS-OCTA and a 73.9% signal loss was observed in SD-OCTA. As a result of this study, it was observed that all signal decreases under the drusen were not associated with the flow reduction of the choriocapillaris but could also be caused by a false-negative flow signal, due to drusen-induced blockage—a “shadowing artefact”. There is not yet any correction software that can totally eliminate these shadowing artifacts. Therefore, the researchers have used retinal pigment epithelium (RPE) elevation maps, to extract these parts, in order to obtain more accurate choriocapillaris vascular density measurements. As a result of this study, it was discovered that there was a significant flow signal loss in choriocapillaris compared with healthy controls, even in areas without drusen in non-neovascular AMD cases [9].

In geographic atrophy, which is the advanced form of non-neovascular AMD, it has been reported that there is a significant loss of choriocapillaris flow under the RPE atrophy area, and flow abnormalities are also observed at the borders of the atrophy [10]. The loss of blood flow around geographic atrophy has been reported to be directly proportional to the growth rate of geographic atrophy [11].

In contrast to conventional dye angiographies, OCTA has provided a visualization of the exact boundaries and intrinsic details of neovascular membranes. Analysis of macular neovascularization (MNV) composition revealed a similar capability to detect capillary pattern, as well as mixed and mature vessels with OCTA and indocyanine green angiography (ICGA), butthe MNV area measured by OCTA was reported to be smaller than ICGA [12]. The most common type of MNV observed in neovascular AMD is type 1 MNV, in which MNV localization occurs between the RPE and the Bruch’s membrane. These lesions, which are observed as fibrovascular pigment epithelial detachments (PED) or late leakage of an undetermined source on fluorescein angiography, can be visualized in the choriocapillaris section of OCTA. According to the data from the COFT-1 study group, the rate of OCTA imaging, supported by structural OCT to detect type 1 MNV, was 85.7% [13]. It has been shown that even type 1 MNVs, which cannot be detected by fluorescein angiography or SD-OCT, can be detected with OCTA [14].

Type 2 MNVs are located above the RPE and are usually seen as smaller but sharper vascular structures than type 1 MNVs in the outer retinal section of OCTA [15]. Type 3 neovascularization originates from the deep capillary plexus. Eyes with unilateral type 3 MNV were also reported to have decreased choriocapillaris perfusion versus fellow non-neovascular eyes, and therefore, it was suggested that outer retinal ischemia might play a role in the type 3 MNV pathogenesis [16].

Many OCTA imaging features, such as the pattern of MVN (medusa, sea fan, glomerular, lacy wheel, etc.), the branching characteristics, anastomosis and loop formation, and the dark halo appearance around the lesion have been described previously [17]. Several researchers have tried determining the lesion activity criteria according to these OCTA pattern characteristics. The pattern of medusa or sea fan, thin capillary structures with numerous branching instead of large thick vessels, the formation of anastomosis and loops, the vessel termini forming peripheral arcades, and the hypointense halo appearance around the lesion, were accepted as activity criteria [18]. If at least three of these five criteria are present, the lesion is considered active. In particular, the existence of a thin capillary structure and arcade formation were identified as the most related criteria with the possibility of exudation [19].

**Figure 2:**
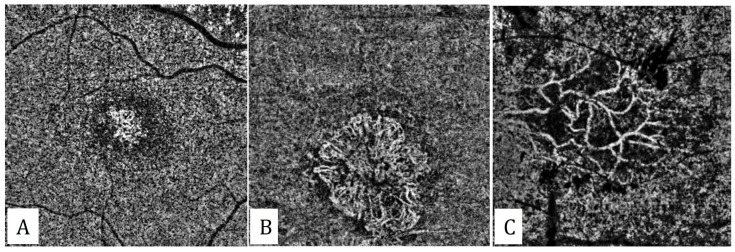
Classification of the maturity of type 1 macular neovascularizations. (**A**) An immature type 1 MNV with abundant number of capillaries. (**B**) A mature type 1 MNV with reduction in the number of capillaries with a well-developed network. (**C**) A hyper-mature type 1 MNV with almost no capillaries.

However, an international panel of experts on the OCTA nomenclature of neovascular AMD has recently introduced new, simplified terminology to provide standardization. According to this latest terminology, maturity of the MNV lesion was categorized as immature, mature, and hyper-mature, according to the density of the capillaries [20]. The MNV lesion was defined as immature in the presence of a dense network of capillary vessels (Figure 2). Mature MNV lesions are those which have a reduced density of capillaries with a well-developed network of vessels. If the MNV lesion was composed of well-delineated vessels with almost no capillaries, it was categorized as hyper-mature. The central trunk was defined as a single large vessel, located anywhere within the lesion, branching into smaller vessels.

**Figure 3:**
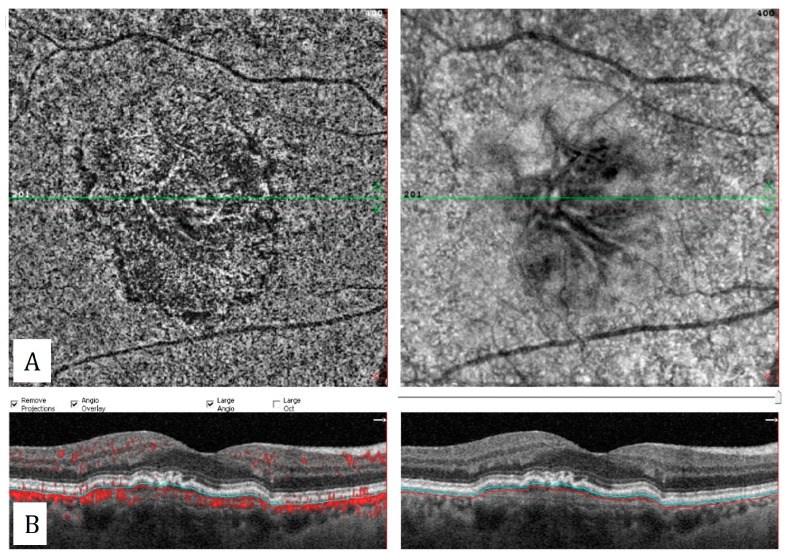
Treatment-naïve quiescent macular neovascularization (MNV). (**A**) Choriocapillaris slab of optical coherence tomography angiography and (**B**) flow data superimposed horizontal B-scan shows well-defined MNV without any exudative feature.

With the increasing use of OCTA in AMD, new concepts such as “quiescent MNV” have emerged. Quiescent type 1 MNV is a novel subclinical subtype of neovascular AMD that does not yet show signs of activation (i.e., exudative symptoms) (Figure 3). The presence of subclinical MNVs has been demonstrated in 14.4% of patients with non-exudative AMD [21]. Visualization of these quiescent, subclinical type 1 MNV lesions by OCTA provides close follow-up of the lesions and facilitates early recognition of exudation. The risk of exudation was 13.6 times greater for eyes with subclinical MNV detected by SS-OCTA, compared with eyes without subclinical MNV after 24 months [22].

Various changes in the morphology of MNV may also occur under anti-vascular endothelial growth factor (anti-VEGF) therapy. The formation of thicker and dilated vessels in the lesion is called “arteriolization”. There are controversial results associated with changes in MNVs receiving anti-VEGF therapies. Minimal changes in the MNV total area and vessel density are reported after anti-VEGF [23]. McClintic et al. reported that active MNV shrank after therapy and then grew again [24]. However, Xu et al. reported the growth of MNVs under anti-VEGF therapy [25]. Bailey et al. reported that growth of 20% per month in silent MNVs carries a risk of exudation [26]. These studies suggest that the growth detected by OCTA is an important criterion for assessing MNV activity.

**Figure 4:**
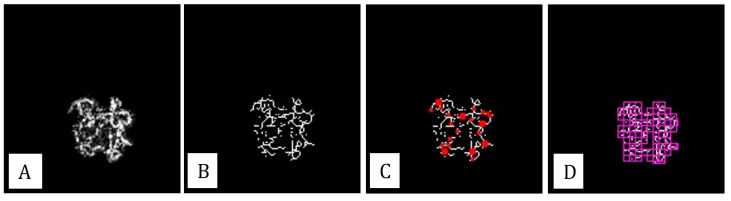
Quantitative analysis of a type 1 MNV lesion with ImageJ version 1.52 u. (**A**) The area of the MNV was manually outlined and measured as 0.792 mm^2^. (**B**) Then, a skeleton model was created by the ImageJ software following Otsu binarization, and the total vessel length of the lesion was measured as 8.3 mm. (**C**) Number of skeleton intersection points (in red color) were calculated as 56. (**D**) Using box contig formula (purple boxes), the fractal dimension and lacunarity values were measured as 1.52 and 0.38, respectively.

New mathematical formulas, fractal dimension and lacunarity, quantitatively enabled the evaluation of MNV characteristics with the help of sophisticated software, such as ImageJ FracLac Plugin, Fractalyse, and MATLAB coding language (Figure 4). The fractal dimension (FD) is a new quantitative parameter that indicates the complexity of the lesion. It has a value between 0 and 2. A higher value refers to the increased complexity of the lesion. Al-Sheikh et al. showed that after anti-VEGF treatment, complexity of the neovascular lesion decreases, due to the loss of small caliber vessels [27]. Lacunarity is a measurement of vascular diversity. High values of lacunarity correspond to heterogeneous networks, whereas low values correspond to the homogeneity of the neovascular lesion. Evaluations with OCTA after intravitreal anti-VEGF injections showed a change in neovascular network from a homogeneous structure with a small branching network to a heterogeneous structure with the loss of capillaries [18].

### 2.2. Pachychoroid Neovasculopathy

Type 1 MNV, that is associated with classical pachychoroid features in the absence of characteristic age-related macular degeneration features, has been defined as pachychoroid neovasculopathy (PNV) [28]. In a study evaluating 88 eyes of 61 central serous chorioretinopathy cases with flat irregular PED appearance, it was reported that MNV was detected with OCTA in one-third of the cases [29].

**Figure 5:**
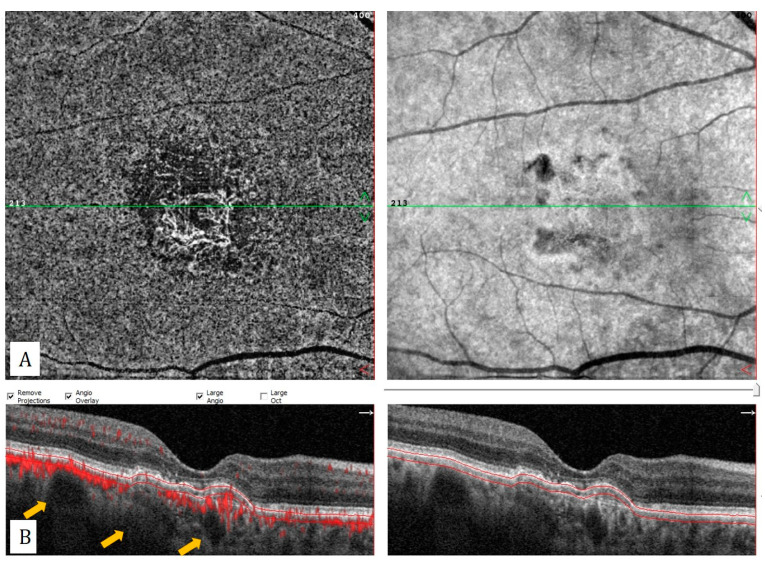
Pachychoroid neovasculopathy: (**A**) Choriocapillaris slab of optical coherence tomography angiography shows macular neovascularization. (**B**) Flow-data-superimposed horizontal B-scan reveals flat irregular pigment epithelial detachment with intrinsic flow signal and dilated Haller vessels (yellow arrows).

OCTA is not affected by choroidal hyperpermeability and can show the exact size of the neovascular membranes in PNV cases (Figure 5). However, in 11.8% of ICGA-proven PNV cases, it was stated that OCTA could not identify MNV due to blockage of fluid or hemorrhage [30]. In contrast, chroroidal hyperpermability and the extend of RPE alterations may limit the use of ICGA for the MNV detection in these cases. Demirel et al. reported a higher sensitivity with OCTA in detection of type 1 MNV, compared to conventional dye angiography in cases with pachychoroid spectrum disease [31].

In the concept of pachychoroid-related disorders, another application of OCTA is the visualization of intervortex venous anastomoses passing through the watershed zone in the macular area. Choroidal slabs of en-face OCTA images could be used for both qualitative and quantitative evaluations of intervortex venous anastomoses [32].

### 2.3. Polypoidal Choroidal Vasculopathy

**Figure 6:**
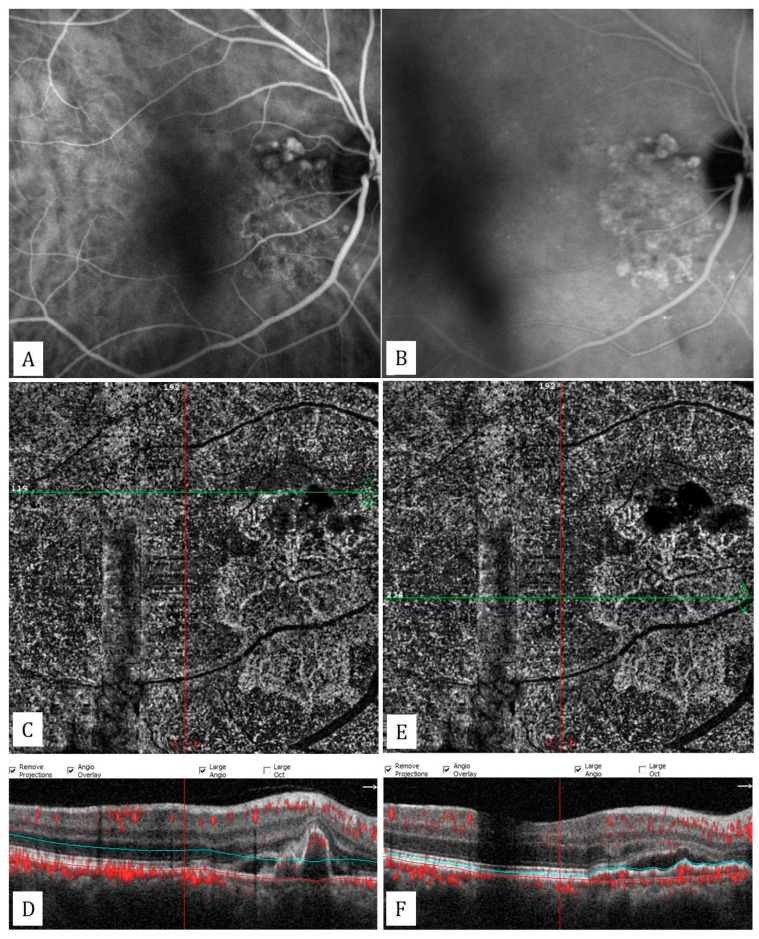
Polypoidal choroidal vasculopathy: (**A**,**B**) Polypoidal structures and branching vascular network on the early and late phases of indocyanine green angiography. (**C**,**D**) Hyporeflective polyps on the choriocapillaris slab of the en-face OCTA and an inverted V shaped polyp with intrinsic flow signal on corresponding flow signal superimposed horizontal B-scan OCT. (**E**,**F**) Branching vascular network on choriocapillaris slab of en-face OCTA and flat irregular pigment epithelial detachment appearance corresponding to branching vascular network on the flow signal superimposed horizontal B-scan OCT.

Polypoidal choroidal vasculopathy (PCV) is characterized by aneurysmal dilatations of the internal choroidal network. Polyps are observed as round structures showing hyper or hypo flow in OCTA [33]. In OCTA imaging, the branching vascular network structure can be visualized better than polypoidal lesions, due to the different intrinsic flow characteristics of polyps (turbulent flow, thrombosis) (Figure 6).

In a study comparing the findings of ICGA and OCTA in PCV, a smaller number of vascular networks, with a larger number of polyps, was reported with ICGA [34]. Additionally, the area of the PCV complex detected by OCTA is wider than the area detected in ICGA in the majority of cases [35]. However, the polyp sizes detected by OCTA are smaller than those of the ICGA [34]. Polypoidal lesions form cluster-like structures in OCTA in 74% of cases, and these cluster-like structures are associated with particularly large-sized polypoidal lesions [36].

### 2.4. Other Macular Neovascularizations

**Figure 7:**
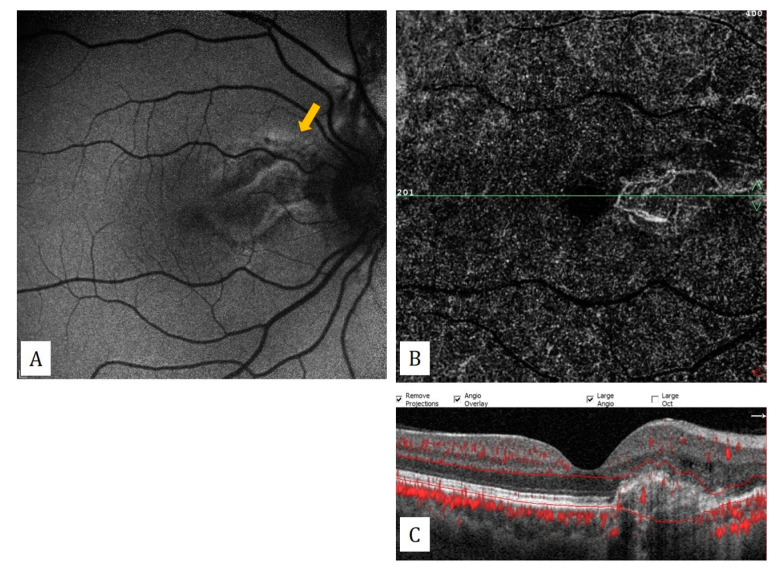
Angioid streaks: (**A**) fundus autofluorescence imaging shows hyperautofluorescent streaks extending from the optic disc (yellow arrow). (**B**) Secondary macular neovascularization on the outer retinal slab of the en-face OCTA. (**C**) The appearance of pigment epithelial detachment causing choroidal excavation on the flow signal superimposed B-scan OCT.

**Figure 8:**
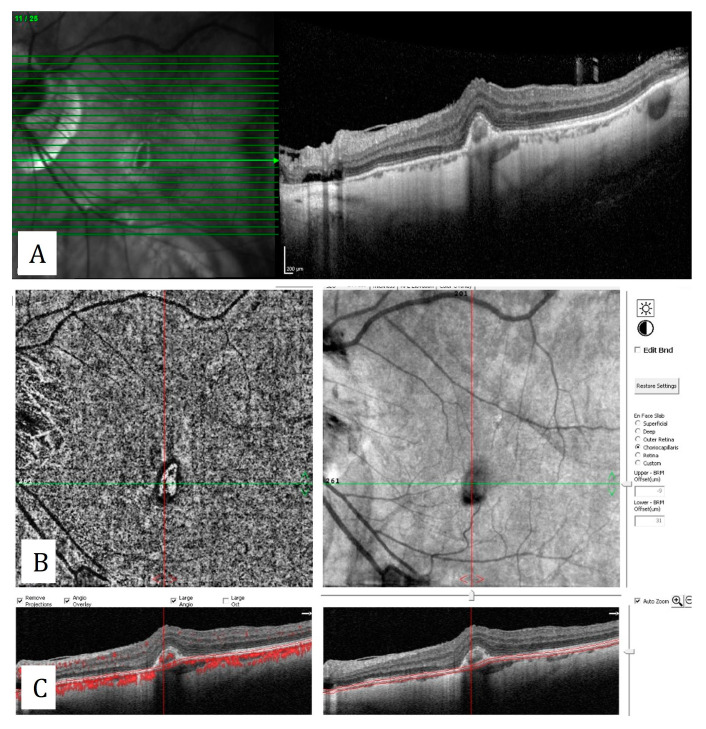
Myopic macular neovascularization: (**A**) Spectral domain optical coherence tomography shows a subfoveal hyperreflective lesion. (**B**) Outer retinal slab of OCTA shows an irregular type 2 macular neovascularization. (**C**) Intrinsic flow signal is observed in the hyperreflective MNV lesion on the flow data superimposed horizontal B-scan.

**Figure 9:**
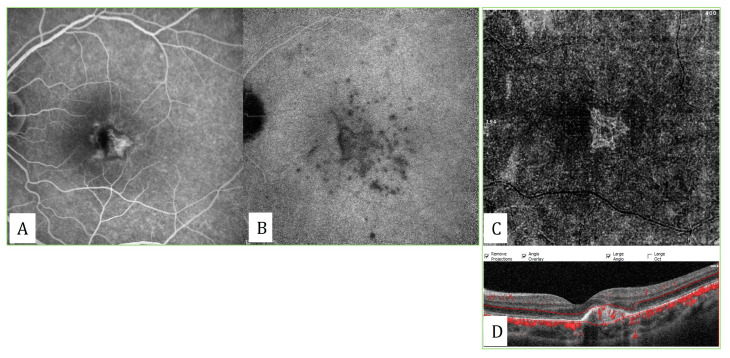
Multimodal fundus images of a patient with inflammatory macular neovascularization (MNV): (**A**) Fluorescein angiography shows centrally located type 2 MNV lesion. (**B**) Indocyanine green angiography shows hypocyanescent spots on the macula. (**C**) Outer retina slab of en-face OCTA reveals the type 2 MNV together with (**D**) intrinsic flow signal on the flow signal superimposed horizontal B-scan OCT.

Macular neovascularization may be idiopathic or may develop secondarily to degenerative causes (angioid streaks (Figure 7), myopic MNV (Figure 8)), hereditary degenerative disorders (vitelliform macular dystrophy), inflammatory pathologies (multifocal choroiditis, MEWDS (Figure 9)), infectious agents (tuberculosis, toxoplasmosis), tumor, and trauma.

OCTA studies in angioid streaks have shown that vascular involvement in the level of choriocapillaris exists even before the development of secondary MNV [37]. In addition, OCTA is an effective method for the detection of MNV in these cases [38].

In the presence of high myopia, even without the accompanying staphyloma or MNV, it is difficult to obtain high-quality OCTA images due to the prolonged axial length. In myopic MNV, the lesion may be difficult to detect because the MNV is smaller than other MNV lesions. Usually, it appears as a hyperreflective material localized on RPE and may not be accompanied by subretinal or intraretinal fluid or is minimal. Therefore, even if OCTA is a part of multimodal imaging in the diagnosis and follow-up of myopic MNV, it should be used in combination with other imaging methods, due to these limitations.

Especially in the presence of inflammatory and infectious MNVs, due to the presence of dense hyperreflective material and inflamed choriocapillaris, conventional dye angiographies may not be able to detect the plaque or hot spot appearance of an MNV. In these cases, it has been shown that the underlying MNVs can be easily visualized with the help of OCTA [39].

OCTA imaging is also a helpful diagnostic tool for hereditary macular and retinal dystrophies. In vitelliform macular dystrophy, MNV lesions may not be visualized in FA or ICGA, due to the blocking effect of vitelliform material. Additionally, in the recirculation phase, the staining of vitelliform material can also avoid the visualization of MNV. OCTA is a useful device for the visualization of MNVs in these cases [4]. Regarding other hereditary dystrophies such as Stargardt or retinitis pigmentosa, it has been reported that the distance between the capillaries increases in the areas above the focal external retinal changes. The region of the choriocapillaris changes has been shown to be smaller than the region of overlying RPE and photoreceptor alteration, suggesting that the vascular loss in these cases was secondary to outer retinal changes [40].

### 2.5. Diabetic Retinopathy

Diabetic retinopathy is one of the microangiopathic complications of diabetes. It has been reported that macular microvascular changes can be visualized with OCTA even when the fundus examination does not yet show any signs of diabetic retinopathy [41].

**Figure 10:**
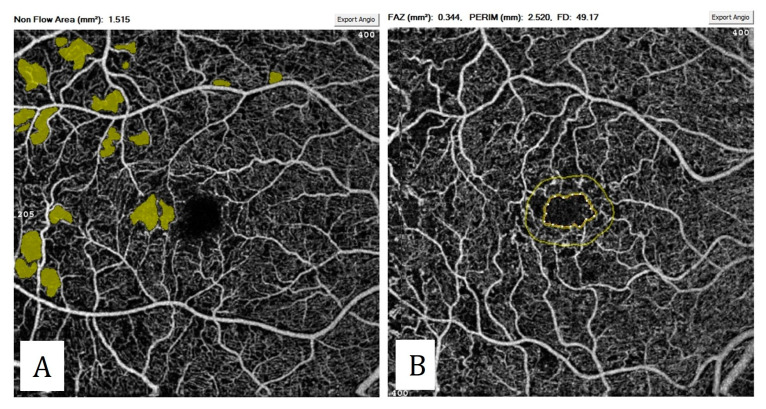
Quantitative measurements in diabetic retinopathy: (**A**) measurement of non-perfused areas. (**B**) Measurement of foveal avascular zone area and perimeter. Note the enlargement and irregularity of the FAZ.

Microaneurysms, capillary non-perfused areas, capillary tortuosity, and dilatation, choriocapillaris abnormalities, IRMA, enlargement of the foveal avascular zone (FAZ), increase in the perifoveal intercapillary space, and retinal and disc neovascularizations can be visualized with OCTA. In addition to these qualitative data, it is capable of quantitatively measuring capillary non-perfusion areas, the FAZ area, the FAZ circularity index, and vascular densities (Figure 10). Among the quantitative parameters, the most frequently studied are the FAZ area measurements. As the severity of diabetic retinopathy increases, the FAZ area enlarges [42]. This enlargement is particularly pronounced in the deep capillary plexus [43].

**Figure 11:**
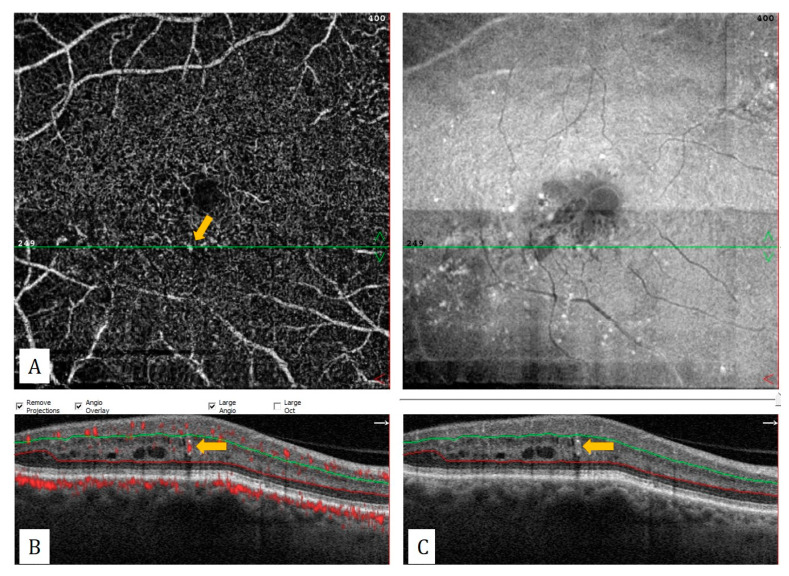
A 6 mm × 6 mm OCTA scan from a patient with diabetic retinopathy demonstrating microaneurysm. (**A**) On deep capillary plexus slab of OCTA, microaneurysm is seen as hyperreflective vascular lesion (yellow arrow). (**B**) Intrinsic flow signal of the microaneurysm (yellow arrow) on flow data superimposed B-scan. (**C**) A thin-walled oval microaneurysm (yellow arrow) is observed on structural B-scan OCT.

Microaneurysms may also be detected by OCTA in DRP (Figure 11). Compared to fluorescein angiography, OCTA may be insufficient to detect microaneurysms since some microaneurysms contain reduced blood flow below the OCTA threshold value. Additionally, another important limitation of OCTA is the auto-segmentation error caused by macular edema. Eyes with DME that respond poorly to anti-VEGF therapy have a significantly larger FAZ area in OCTA and more microaneurysms in the deep capillary plexus, compared to eyes that respond well to anti-VEGF therapy [44]. Another important limitation to be considered is that the edematous cystoid spaces can be confused with the exact ischemic areas. The cystoid spaces in DME appear devoid of flow on the OCTA and are oblong in shape with smooth borders that do not follow the distribution of surrounding capillaries, whereas areas of capillary nonperfusion have a grayer hue and irregular borders [45].

Proliferative diabetic retinopathy, though OCTA does not provide vascular leakage information, may be useful in the differentiation of IRMA and NVE. Especially on the flow data superimposed B-scans, the flow signal is located under the internal limiting membrane in IRMA. IRMA, which is considered a risk factor for developing proliferative diabetic retinopathy, has been shown to be localized adjacently to preretinal NVE areas in OCTA [46].

Nowadays, it is possible to generate composite OCTA images, providing a wide-field view up to 80°, using the montage tool of the devices. However, the field of view is still significantly less than the 200° provided by ultra-wide field (UWF) FA systems and wide-field OCTA imaging is the most promising technique for the near future. The mean percentages of correct neovascularization grading were reported to be very similar using SS-OCTA with B-scans (87.8%) and using FA 86.2% (*p* = 0.92) [47]. Couturier et al. observed that the detection rate of nonperfusion areas was higher with swept-source, wide-field OCTA than with UWF FA [48].

### 2.6. Retinal Vein Occlusions

**Figure 12:**
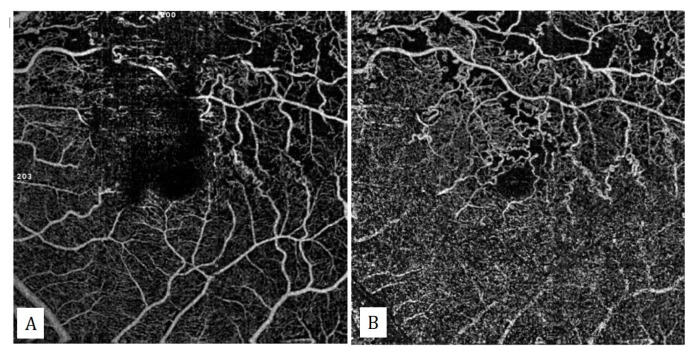
En-face OCTA image showing superficial (**A**) and deep (**B**) capillary plexuses of a patient with superiotemporal branch vein occlusion in his left eye. Capillary non-perfusion areas and retinal shunt vessels are seen. Retinal shunt vessels are especially pronounced in the deep capillary plexus.

Retinal vein occlusions are the second-most-common retinal vascular disease after diabetic retinopathy. OCTA can be used to evaluate both branch retinal vein occlusions (BRVO), and central retinal vein occlusions (CRVO). In these cases, increased venous folding, capillary nonperfusion areas, intraretinal shunt vessels, and emerging neovascularizations can be easily visualized with OCTA (Figure 12).

It has been shown that there is a decrease in the vascular density of both the superficial and deep capillary plexi in venous occlusions. However, the amount of microvascular occlusion is more pronounced in the deep vascular plexus compared to the superficial plexus, and appears to be associated with the development of paracentral acute middle maculopathy [49]. In a study evaluating the effect of FAZ changes on visual prognosis in CRVO cases, it was reported that the FAZ was enlarged in both the superficial and the deep capillary plexus in OCTA, and there was a significant correlation between the FAZ area and visual acuity in cases without macular edema [50].

The most important limitation of OCTA in the imaging of venous occlusions is suboptimal segmentation due to intraretinal edema or retinal thinning. In addition, peripheral retinal ischemia cannot be evaluated due to the limited field of view of OCTA. Although a study comparing fluorescein angiography and OCTA showed high compatibility between the two methods for detecting non-perfusion areas, it was shown that 46% of the eyes had nonperfusion areas and 32% had retinal neovascularization in the area outside the 8 × 8 mm OCTA screening area [51]. However, this limitation will soon be eliminated with the promotion of wide-field OCTA systems. For the detection of marked nonperfusion, widefield OCTA had been reported to have a sensitivity of 100% and a specificity of 64.9% [52].

### 2.7. Retinal Artery Occlusion

**Figure 13:**
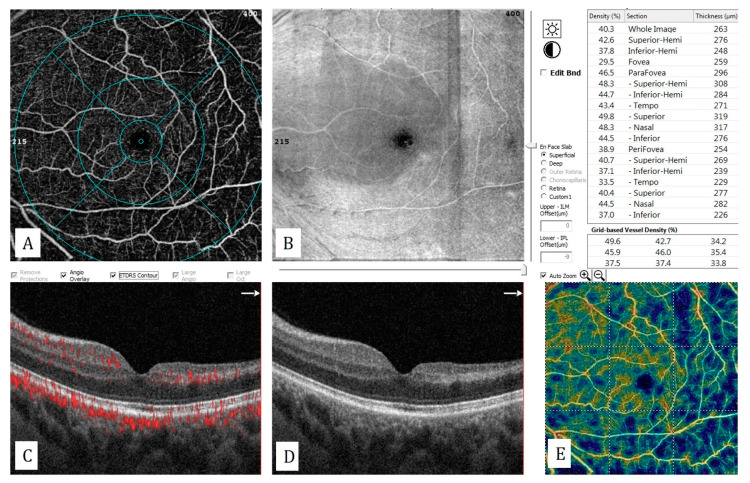
Central retinal artery occlusion with sparing of the cilioretinal artery: (**A**) Decrease in the number of temporal capillaries on the en-face OCTA. (**B**) Hyperreflectivity of retina corresponding the affected area. (**C**) Hyperreflectivity of the inner nuclear layer, temporal to the fovea on the flow-signal-superimposed B-scan OCT. (**D**) Hyperreflectivity of the inner nuclear layer and thinning of the inner retinal layers, temporal to the fovea on structural B-scan OCT. (**E**) The decrease in vascular density on grid-based vascular density analysis.

Retinal artery occlusion is an ophthalmic emergency. In the literature, there are a limited number of case reports or case series detailing the use of OCTA in retinal artery occlusion. In acute retinal perfusion limitations, OCTA imaging has been proven to reveal the precise boundary of the ischemic retinal area, especially on the deep capillary plexus (Figure 13) [53].

### 2.8. Idiopathic Macular Telangiectasia Type 2

**Figure 14:**
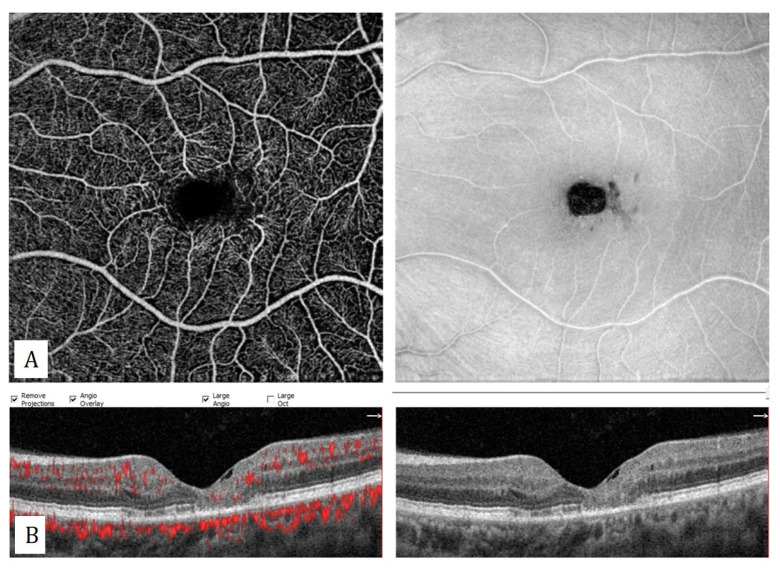
Idiopathic macular telangiectasia type 2. (**A**) Enlargement and irregularity of the foveal avascular zone, right-angled venules, mild telangiectatic changes with decreased vessel density in superficial capillary plexus. (**B**) Hyporeflective cavity, atrophy of outer retinal layers, and internal limiting membrane drape on the flow-signal superimposed B-scan OCT.

Idiopathic macular telangiectasia type 2 (MacTel 2 or perifoveal telangiectasia) is a macular disorder characterized by Muller cell degeneration and loss. The vascular changes in the MacTel type 2 include the enlargement of the FAZ, increase in the distance between capillaries, capillary losses, telangiectatic changes, and the formation of superficial and deep capillary plexi anastomosis (Figure 14).

An OCTA-based staging system was described by Toto et al. [54]. The authors classified the extent of the vascular changes on OCTA according to the lateral extension of vascular anomalies, using the foveal center as the main landmark: temporal to the fovea, spread nasally, circumferentially, and outer retinal neovascularization [54]. While the superficial capillary plexus is usually preserved in the early stage of the disease, early changes originate from the deep capillary plexus, especially the temporal parafoveal area [55]. The right-angled retinal venules, extending from the epiretinal surface to the deep capillary plexus, are also an additional imaging feature described with OCTA [56].

In the advanced stages, vascular abnormalities also affect the outer retina, which is normally avascular. Neovascularization develops in the subretinal space. Anastomosis may occur between subretinal neovascularization and choroidal circulation. Epiretinal neovascularizations are another newly defined OCTA finding [57]. In these cases, intraretinal pigment formation, and severe retinal thinning have been reported.

### 2.9. Other Vascular Diseases of the Retina

Microvascular abnormalities in sickle cell disease affect both superficial and deep capillary plexi. OCTA has higher sensitivity than FA for the detection of macular microangiopathies in asymptomatic patients [58]. These findings can be seen at any stage of retinopathy, but OCTA findings are more pronounced in the presence of the SC genotype or in proliferative retinopathy [59].

If deep capillary ischemia affects the inner nuclear and external plexiform layers, it is called paracentral acute middle maculopathy (PAMM). If it influences the outer plexiform and the outer nuclear layers, then it is called acute macular neuroretinopathy (AMN). Although these two clinical conditions were initially classed as two different types of the same disease, they are now considered different clinical entities. The deep vascular complex consists of two different capillary networks: the intermediate and the deep capillary plexus. If the intermediate plexus is affected, PAMM develops; if the deep capillary plexus is affected, AMN develops. These two clinical pictures are differentiated from each other according to the retinal layer, where the hyperreflective band is located in the structural B-scan images, or where nuclear atrophy develops. In OCTA, vascular density reduction is observed in the deep capillary plexus [60].

Susac syndrome is a microangiopathy that affects the arterioles of the brain, retina, and cochlea. It may cause repetitive retinal artery occlusions. In OCTA, a decrease in vascular density has been shown in both superficial and deep capillary plexi, and partial improvement in vascular densities has been reported under treatment [61].

## 3. Conclusions

OCTA has gained widespread use, both in daily practice and for clinical research purposes. It has been shown to be very useful, especially in the diagnosis and follow-up of many retinal vascular diseases and MNVs. Although it has not yet completely replaced conventional dye angiographies, the limitations that restrict its clinical use are being overcome with the advancements made in this developing technology.
